# Adesão e Desempenho na Reabilitação Cardíaca: O Papel da Inovação Tecnológica

**DOI:** 10.36660/abc.20250337

**Published:** 2025-07-01

**Authors:** Sevda Ece Kizilkilic, Juliana Goulart Prata Oliveira Milani, Gerson Cipriano, Paul Dendale, Mauricio Milani

**Affiliations:** 1 Hasselt University Faculty of Medicine and Life Sciences Hasselt Flanders Bélgica Hasselt University – Faculty of Medicine and Life Sciences, Hasselt, Flanders – Bélgica; 2 Jessa Hospital Heart Centre Hasselt Hasselt Bélgica Jessa Hospital – Heart Centre Hasselt, Hasselt – Bélgica; 3 Ghent University Faculty of Medicine and Health Sciences Gent Bélgica Ghent University, Faculty of Medicine and Health Sciences, Gent – Bélgica; 4 Universidade de Brasília Programa de Pós-Graduação em Ciências e Tecnologias da Saúde Brasília DF Brasil Universidade de Brasília – Programa de Pós-Graduação em Ciências e Tecnologias da Saúde, Brasília, DF – Brasil; 5 Hasselt University Faculty of Rehabilitation Sciences Hasselt Flanders Bélgica Hasselt University – Faculty of Rehabilitation Sciences, Hasselt, Flanders – Bélgica

**Keywords:** Reabilitação Cardíaca, Telemedicina

A reabilitação cardíaca (RC) desempenha um papel central na prevenção secundária da doença arterial coronariana (DAC) e é classificada como uma recomendação Classe IA nas diretrizes clínicas.^1^ No entanto, apesar de seus benefícios comprovados, as taxas de participação permanecem desanimadoramente baixas.^2^ Diante disso, a telerreabilitação (TR) tem ganhado atenção como uma alternativa prática, oferecendo melhorias clínicas semelhantes às dos programas tradicionais baseados em centros especializados.^3^

Paralelamente, a integração da inteligência artificial (IA) tem despertado crescente interesse na área da saúde devido ao seu potencial em diferentes contextos clínicos. Entre as diversas aplicações da IA, o processamento de linguagem natural (PLN) está sendo cada vez mais utilizado em diversas áreas médicas. No entanto, sua aplicação no contexto da CR para interpretar as experiências subjetivas dos pacientes ainda é limitada.

O estudo de Saklica et al.^4^ adota uma abordagem inovadora ao combinar dados clínicos estruturados com uma análise baseada em PLN de *feedback* aberto dos pacientes, oferecendo uma nova perspectiva sobre como a tecnologia pode enriquecer tanto a entrega quanto a avaliação da CR.

O estudo^4^ foi um ensaio clínico randomizado, incluindo 52 pacientes com DAC estável, divididos em três grupos: (1) grupo de TR, com sessões remotas supervisionadas de exercícios, (2) grupo de aplicativo móvel, utilizando um aplicativo de *smartphone* para exercícios guiados em casa, e (3) um grupo controle, que recebeu apenas orientações gerais sobre atividade física. Ao longo de 12 semanas, os participantes dos grupos TR e de app móvel treinaram três vezes por semana, combinando treinamento aeróbico e de resistência, enquanto o grupo controle seguiu recomendações gerais de atividade física, sem um plano estruturado.

Ambos os grupos de intervenção apresentaram melhoras significativas na capacidade de exercício, avaliada utilizando-se o *Incremental Shuttle Walk Test* (ISWT), em comparação com o grupo controle (p=0,001). Essas melhorias excederam a diferença mínima clinicamente importante para o ISWT, indicando um ganho funcional significativo. Os achados estão alinhados com estudos anteriores que demonstram que a TR pode ser tão eficaz (ou até mais) que a RC baseada em centros.^3,5^

A adesão também foi substancialmente maior nos grupos de intervenção: 100% no grupo TR e 80% no grupo de app móvel MAG, em comparação a apenas 30% no grupo controle (p<0,001). Esse aumento na adesão à TR está de acordo com vários estudos que mostram que a frequência à CR é maior com TR do que com programas realizados em centros, provavelmente devido à sua conveniência e flexibilidade.^5-10^

Um ponto interessante deste estudo é o uso de PLN para analisar as reflexões escritas dos pacientes sobre sua reabilitação. Embora a IA tenha sido aplicada em diversas áreas da saúde, seu uso para avaliar as experiências dos pacientes na RC ainda é raro. Neste estudo, os participantes forneceram comentários abertos sobre sua experiência de reabilitação, que foram analisados por um modelo de PLN BERT (*Bidirectional Encoder Representations from Transformers*) ajustado. A análise identificou temas recorrentes, como motivação, fadiga e satisfação, de maneira consistente e estruturada. Curiosamente, o estudo relata uma forte correlação positiva (r = 0,75; p < 0,001) entre o sentimento do paciente e sua melhoria na distância do ISWT, sugerindo que pacientes com maior satisfação e experiência positiva tendem a apresentar maiores melhorias na capacidade de exercício.

Apesar dos pontos fortes, o estudo também apresenta algumas limitações. Primeiro, os grupos seguiram programas de treinamento diferentes. O grupo de TR participou de sessões estruturadas com exercícios de resistência e calistenia. O grupo de app móvel seguiu exercícios semelhantes por meio de vídeos pré-gravados, enquanto o grupo controle não seguiu nenhum programa estruturado. Essas diferenças vão além do modo de entrega e podem explicar parcialmente os melhores resultados nos grupos de intervenção. Segundo, os métodos de monitoramento da adesão variaram entre os grupos: no TR e no grupo app móvel, a adesão foi medida objetivamente, enquanto no grupo controle, foi avaliada por meio de autorrelato. Essa diferença na medição pode ter introduzido viés, pois o autorrelato é propenso à superestimação e à subnotificação. Embora a análise de IA tenha sugerido que alguns pacientes do grupo controle podem ter superestimado seus níveis de atividade, é possível que outros não tenham registrado com precisão sua participação, levando a uma subestimação da adesão.

Finalmente, apesar de o uso de PLN ter sido inovador, trata-se ainda de uma aplicação em estágio inicial, testada em uma amostra pequena. É necessária validação externa em coortes maiores e em populações mais diversas para avaliar sua generalização, uma vez que o *feedback* dos pacientes pode variar dependendo da cultura, idioma e forma como as perguntas são feitas. Esses fatores influenciam como os modelos de PLN interpreta as respostas. Um modelo ajustado para entrada em turco, como no estudo, pode não se traduzir automaticamente para outros contextos de saúde.

O estudo de Saklica et al.^4^ traz novas perspectivas sobre o uso de ferramentas digitais na RC. As melhorias na capacidade de exercício e na adesão em ambas as abordagens baseadas em tecnologia sugerem que as intervenções digitais podem apoiar a prevenção secundária em pacientes com DAC estável. O uso de PLN para analisar o feedback dos pacientes é um aspecto inovador e reflete uma tendência mais ampla de integrar a experiência do paciente na avaliação dos desfechos.

Ao mesmo tempo, várias limitações, como diferenças no conteúdo dos exercícios e nos métodos de medição da adesão, indicam que os resultados devem ser interpretados com cautela. Por fim, embora o uso de PLN seja promissor, ainda está em estágio inicial e precisa de mais validação em populações maiores e mais diversas.
