# Supplementary material for: Adesão e Desempenho na Reabilitação Cardíaca: O Papel da Inovação Tecnológica
Source: Arq Bras Cardiol. 2025 Jul 1;122(6):e20250337. [Article in Portuguese] doi: 10.36660/abc.20250337 (PMC12269902; doi:10.36660/abc.20250337)
Supplement: Translation

# Translation: Adherence and Performance in Cardiac Rehabilitation: The Role of Technological Innovation

**DOI:** 10.36660/abc.20250337i

**Authors:** Sevda Ece Kizilkilic, Juliana Goulart Prata Oliveira Milani, Gerson Cipriano, Paul Dendale, Mauricio Milani

**Affiliations:** 1 Hasselt Flanders Belgium Hasselt University – Faculty of Medicine and Life Sciences, Hasselt, Flanders – Belgium; 2 Hasselt Belgium Jessa Hospital – Heart Centre Hasselt, Hasselt – Belgium; 3 Gent Belgium Ghent University, Faculty of Medicine and Health Sciences, Gent – Belgium; 4 Brasília DF Brazil Universidade de Brasília – Programa de Pós-Graduação em Ciências e Tecnologias da Saúde, Brasília, DF – Brazil; 5 Hasselt Flanders Belgium Hasselt University – Faculty of Rehabilitation Sciences, Hasselt, Flanders – Belgium

**Keywords:** Cardiac Rehabilitation, Telemedicine

Cardiac rehabilitation (CR) plays a central role in secondary prevention of coronary artery disease (CAD) and is classified as a Class IA recommendation in guidelines.^1^ Yet, despite its proven benefits, participation rates remain disappointingly low.^2^ In response, telerehabilitation (TR) has gained attention as a practical alternative that offers similar improvements in clinical outcomes to traditional, center-based programs.^3^

In parallel, artificial intelligence (AI) integration is growing in interest in healthcare due to its potential in different clinical settings. Among the various AI applications, natural language processing (NLP) is increasingly used across medical domains. However, its application in the context of CR to interpret patients’ subjective experiences remains limited.

The study by Saklica et al.^4^ takes a novel approach by combining structured clinical data with NLP-based analysis of open-ended patient feedback, offering a perspective on how technology may enrich both the delivery and evaluation of CR.

The study^4^ was a randomized controlled trial including 52 patients with stable CAD, divided into three groups: a TR group (TRG) with supervised remote exercise sessions, a mobile application group (MAG) using a smartphone app for guided home exercise, and a control group receiving only standard physical activity advice. Over 12 weeks, the TRG and MAG participants trained three times per week with a mix of aerobic and resistance training, while the control group followed general activity recommendations without a structured plan.

Both intervention groups showed notable improvements in exercise capacity, assessed using the Incremental Shuttle Walk Test, compared to the control group (p=0.001). These improvements exceeded the minimal clinically important difference for ISWT, indicating a meaningful gain in functional fitness. The findings are in line with previous studies showing that TR can be as effective (or even more) than center-based CR.^3,5^

Adherence was also substantially higher in the intervention arms: 100% in the TRG and 80% in the MAG, versus just 30% in the control group (p<0.001). This greater increase in adherence to TR aligns with multiple studies that have shown that CR attendance is higher with TR than with center-based CR, likely due to its convenience and flexibility.^5-10^

An interesting element of this study is the use of NLP to analyze patients’ written reflections on their rehabilitation. While AI has been applied in various areas of healthcare, it has rarely been used to assess patient experiences in CR. In this study, participants provided open-ended comments about their rehabilitation experience, which were analyzed using a fine-tuned BERT (Bidirectional Encoder Representations from Transformers) NLP model. The analysis captured recurring themes, such as motivation, fatigue, and satisfaction, in a consistent and structured way. Interestingly, the study reports a strong positive correlation (r = 0.75, p < 0.001) between patient sentiment and their improvement in ISWT distance, suggesting that patients with higher satisfaction and positive experience tended to show greater improvements in exercise capacity.

Despite its strengths, the study also has a few limitations. First, the groups followed different training programs. The TRG group participated in structured sessions with resistance and calisthenics exercises. In contrast, the MAG group followed similar exercises using pre-recorded videos, while the control group did not follow any structured program. These differences go beyond delivery mode and may partly explain the better outcomes in the intervention groups. Second, adherence monitoring methods varied across groups: it was objectively measured in the TRG and MAG groups, whereas in the control group, adherence was assessed through self-reporting. This difference in measurement may have introduced bias, as self-reporting is prone to overestimation and underreporting. Interestingly, the AI analysis suggests that some patients in the control group may have overestimated their activity levels, but it is also possible that others failed to accurately record their participation, potentially leading to underestimation of adherence.

Finally, while using NLP is innovative, it remains an early-stage application tested in a small sample. More external validation in larger cohorts is needed in more diverse populations to assess generalizability, since patient feedback can differ depending on the culture, language, and the way questions are asked, which can all influence how NLP models interpret responses. A model fine-tuned on Turkish-language input, as in this study, may not automatically translate well to other healthcare settings.

The study of Saklica et al.^4^ provides new insights into the use of digital tools in CR. The improvements in exercise capacity and adherence in both technology-based CR suggest that digital interventions can support secondary prevention in stable CAD patients. The use of NLP to analyze patient feedback is a novel aspect and reflects a broader shift toward integrating patient experience into outcome evaluation.

At the same time, several limitations, such as differences in exercise content and adherence measurements, mean that the results should be interpreted with care. Lastly, while the use of NLP is promising, it remains in an early stage and needs further validation in larger and more diverse populations.
